# Diagnosis of Idiopathic Pulmonary Fibrosis: Differential Diagnosis

**DOI:** 10.3390/medsci6030073

**Published:** 2018-09-04

**Authors:** Myriam Aburto, Inmaculada Herráez, David Iturbe, Ana Jiménez-Romero

**Affiliations:** 1 Department of Respiratory Medicine, Galdakao Hospital, 48960 Galdako, Spain; 2 Department of Radiology, University Hospital of León, 24071 León, Spain; iherraez@saludcastillayleon.es; 3 Department of Respiratory Medicine, Marques de Valdecilla University Hospital, 39008 Santander, Spain; david.iturbe@scsalud.es; 4 Department of Respiratory Medicine, El Bierzo Hospital, 24404 Ponferrada, Spain; anajimenezr@saludcastillayleon.es

**Keywords:** idiopathic pulmonary fibrosis lower case, usual interstitial pneumoniae, idiopathic interstitial pneumonia, surgical lung biopsy, transbronchial cryobiopsy, diagnosis, high-resolution computed tomography, multidisciplinary team

## Abstract

Idiopathic pulmonary fibrosis (IPF) is a chronic, progressive, and fibrotic interstitial lung disease of unknown origin with a characteristic imaging and histologic pattern called usual interstitial pneumonia (UIP). The diagnosis of IPF is a complex procedure that requires the support of various specialists, who must integrate clinical, radiological, and histological data. The multidisciplinary team (MDT) has become the new gold standard to diagnose and manage the disease, increasing the accuracy and agreement of the diagnosis between different centers. It is mandatory to exclude nonspecific interstitial pneumonia or other diseases that can cause the UIP pattern, particularly drugs or exposure diseases, including chronic hypersensitivity pneumonitis or systemic autoimmune disease. The role of the MDT is also to decide who could need a biopsy or to review patient diagnoses at regular intervals in those with additional information or unexpected evolution. This review provides updated information to achieve a proper IPF diagnosis.

## 1. Diagnosis of Idiopathic Pulmonary Fibrosis

Idiopathic pulmonary fibrosis (IPF) is a chronic, progressive, and fibrotic interstitial lung disease of unknown origin with a characteristic imaging and histological pattern called usual interstitial pneumonia (UIP). IPF is of particular concern, because its prognosis is very poor, with a median survival rate of three years even in recent publications [1].

Early intervention is crucial to diagnosing the disease, because it is often misdiagnosed as heart failure or chronic obstructive pulmonary disease; therefore, patients spend around two years [2] without a proper evaluation in an interstitial lung disease (ILD) center. The initial identification and assessment of possible ILD in primary care is a key aspect. NICE (National Clinical Guideline Centre) clinical guidelines in 2013 [3] identified a group of patients at risk as follows: over 45 years of age, persistent breathlessness on exertion, persistent cough, bilateral inspiratory crackles when listening to the chest, and clubbing of the fingers. People with these clinical features should be referred to secondary care to establish a diagnosis and to enable the initiation of appropriate clinical management. High-resolution computed tomography (HRCT) of the chest is mandatory in order to assess if ILD is present and, if so, to begin the differential diagnosis.

The published information on IPF from this decade has envisioned a radical change in its management and treatment. Since the publication of the revised diagnostic criteria for IPF [4] by the American Thoracic Society (ATS), European Respiratory Society (ERS), Japanese Respiratory Society (JRS), and Latin American Thoracic Association (ALAT) in 2011, positive results have been obtained in large-cohort clinical trials treating IPF with two drugs for the first time. Relevant information about the clinical course, comorbidities, and new diagnostic tools have introduced new nuances into the diagnostic process of the disease. The Fleischner Society [5] has noted these changes and has published a new management proposal recently.

**Figure 1:**
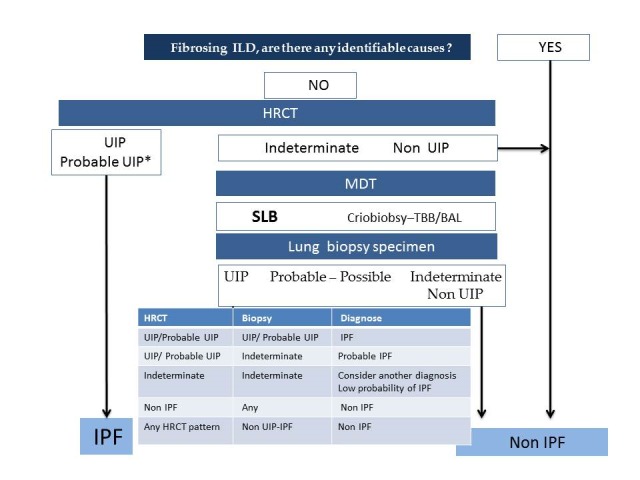
Flow chart to diagnose idiopathic pulmonary fibrosis (IPF). ILD: interstitial lung disease; HRCT: high-resolution computed tomography; UIP: usual interstitial pneumonia; MDT: Multidisciplinary team; SLB: surgical lung biopsy; TBB: transbronchial forceps biopsy; BAL: bronco alveolar lavage; *: patient with “probable UIP pattern” in high-resolution computed tomography (HRCT) and clinical high suspicion of IPF (patient over 60 years, smoking history, and unknown etiology do not require a biopsy).

The diagnosis of IPF is a complex procedure that requires the support of various specialists, who must integrate clinical, radiological, and histological data, sometimes with uncertain or conflicting information. Therefore, such multidisciplinary teams (MDTs) have become the new gold standard to diagnose and manage the disease [4,5]. The MDT should at least be composed of a pulmonologist, a thoracic radiologist, and an expert ILD pathologist [3]. The information added by a thoracic surgeon or a rheumatologist can also be relevant in deciding the point of biopsy or ruling out a systemic autoimmune disease (SAD). The pulmonologist provides the clinical context, exploring the patient’s history and physical examination, the radiologist interprets the pattern present in HRCT images of the chest, and approximately 30% of patients may need a lung biopsy sample [6] that must be carefully examined by an expert in ILD pathology. Figure 1 shows the flow chart of the diagnostic procedure.

Practical issues concerning the MDT are the following [5]: The MDT should meet regularly—weekly, biweekly, or at least monthly, depending on the volume of patients. In experienced groups with a high volume of patients, those with typical features of IPF (patients with clinical context of IPF: older than 60 years, absence of clinically significant environmental or medication exposure, no evidence of connective tissue disease, and UIP pattern in HRCT) might not require review. Selected patients might also be re-reviewed on follow-up. If some members of the team are short of experience, then it should be mandatory to arrange a linkage to an experienced group. In some patients with undetermined features who lack surgical biopsy specimens because of concomitant comorbidities or patient declination, bronchoalveolar lavage or transbronchial biopsy must be performed in order to add as much information as possible [5]. In this case, it is possible to provide a working diagnosis with consideration of the patient’s age, sex, smoking status, and longitudinal disease behavior. This working diagnosis must include the level of diagnostic accuracy by adding terms like “confident” or “provisional with high or low confidence”, and the diagnosis should be reviewed at regular intervals, because it might change over time.

The MDT reduces diagnostic imprecision and increases the accuracy of diagnosis by combining information from all three domains. Works published from a single hospital reveal that 41.9% of previous diagnoses were changed and that in 81.9% of previously considered indeterminate cases, an MDT was able to reach a proper diagnosis [7]. Despite these figures, the MDT has not been formally validated and lacks the incorporation of a standardized model of validation that is applied to most other health diagnostic procedures. MDT comparisons [8] from highly expert ILD centers have shown that agreement on IPF as a first-choice diagnosis was good (weighted κ = 0.71), but there was poor agreement on the diagnosis of other fibrosing ILD, such as chronic hypersensitivity pneumonitis (κ = 0.24) or idiopathic non-specific interstitial pneumonia (κ = 0.25). The MDT was able to increase the diagnostic agreement and reproducibility in less experienced centers [9].

### 1.1. Clinician Assessment

Any patient with an ILD in HRCT, should undergo a narrow clinical and physical examination to exclude exposure-related conditions or SAD. Familial pulmonary fibrosis must also be investigated. The clinical probability of IPF increases if the patient is older than 60 years, male, and has a history of cigarette smoking but requires exclusion of alternative causes of fibrosing ILD. The main diagnostic alternatives are covered in other paragraphs in this article. The clinical assessment [3,4] includes a wide range of questions about domestic or work environment issues but also about free time activities. A structured questionnaire may be helpful to obtain all the needed information. It is necessary to inquire about the following: water damage; presence of air conditioning; humidifiers; hot tubs; visible mold in the home, basement, or workplace; possible contact with birds or feathers, including bedding clothes, pillows cushions, or armchairs; agricultural exposure to moldy hay, animal feeds, or composting; using steam baths or swimming pools; or exposure to any moldy material in free time. Care must be taken about possible current or previous occupational exposure to asbestos, coal, silicate, or isocyanate. In suspected exposures, serum precipitins or specific immunoglobulin G (IgG), air cultures, or bronco alveolar lavage (BAL) cell count would provide additional information [10,11]. The main limitation is the lack of a reliable clinical guide to diagnose chronic hypersensitivity pneumonitis (HP), and in up to 50% of confirmed cases of HP with lung biopsy, the antigen remains unidentified [12].

A systematic assessment for SAD is necessary. The HRCT UIP pattern appears more often in systemic sclerosis, rheumatoid arthritis, or Sjögren syndrome but can be present in any other SAD. For some patients, fibrosing lung disease could be the first sign of an immune condition; therefore, it is especially important to inquire about signs or symptoms suggestive of an autoimmune disease. In the absence of relevant signs, serological tests—including at least antinuclear antibodies (ANA) antibodies and pattern, anti-DNA, anti-neutropil cytoplasmic antibody (ANCA), rheumatoid factor (RF), and Anti-citrullinated protein (CCP) antibodies, SS-A, SS-B, anti Jo-1, creatine-kinase, and myohemoglobin—should be performed in all patients. If available, other anti-synthetase antibodies, such as anti-PL-12 and anti-PL-7, could be helpful to exclude inflammatory myopathies that accompany the UIP pattern. In the case of any clinical suspicion, the serological tests should be performed. For those who do not meet the criteria to be diagnosed with any SAD, the possibility of suffering “interstitial pneumonia with autoimmune features” must keep in mind [13].

Currently, there are not enough data to perform an active search for relatives among patients with IPF, and it is not justifiable to perform a genetic test in all IPF patients. French guidelines [14] recommend researching clinical signs of genetic causes in patients with concomitant hepatic, cutaneous, mucosal, or hematological abnormalities of unknown origin, particularly in those under 50 years.

Radiological involvement caused by a drug is also common. The patient should be asked about previous treatment and duration, including radiotherapy or chemotherapy.

There is still no biological marker that allows IPF to be diagnosed. These serological biomarkers can be found in different fibrosing ILD and are more associated with the evolution and prognosis of the disease, and their position in clinical practice has not been clarified.

### 1.2. Radiological Diagnosis of Idiopathic Pulmonary Fibrosis

The presence of IPF can be suspected on a chest X-ray when a reticular pattern, predominantly basal, with or without cystic airspaces, is observed, especially if lung volumes are reduced. However, these features can also appear in other fibrotic diseases (e.g., chronic hypersensitivity pneumonitis, systemic autoimmune disease-associated pulmonary fibrosis, or stage IV sarcoidosis) and in non-fibrotic ones (e.g., bronchiectasis, infections, or bullous emphysema).

Therefore, it is necessary to perform a volumetric multidetector HRCT [15] in patients with clinic and/or chest X-ray suspicion of IPF. The volumetric HRCT should be done with the patient in decubitus supine, keeping apnea in forced inspiration and usually is completed with 3–4 high-resolution sequential slices at the end expiration. It is imperative to obtain high-quality images, thinner than 2 mm and with a high-spatial resolution reconstruction algorithm. Sometimes, it may be necessary to perform volumetric HRCT in decubitus prone if the images in supine are not of sufficient quality to properly evaluate alterations in the posterior lower lobe areas to differentiate lung disease from normal dependent atelectasis.

For very dyspneic patients who are unable to keep inspiration apnea during image acquisition, the volumetric HRCT will be done in decubitus lateral, asking the patient to hold a shallow breath. As the non-declined lung inspires, it usually shows limited breath artifacts, making it very useful to evaluate the lung affectation pattern and its distribution. As the declined lung expires, it can be used to evaluate the air trapping.

#### 1.2.1. High-Resolution Computed Tomography Patterns

**Figure 2:**
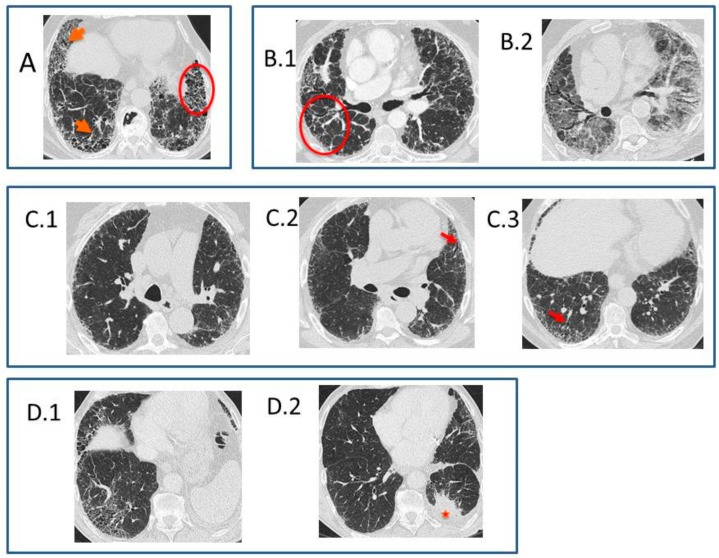
HRCT in IPF. (**A**) Typical UIP CT pattern. HRCT image: honeycombing (circle) and reticular opacities with peripheral traction bronchiectasis (arrows), with peripheral subpleural and basal-predominant distribution. (**B**) CT pattern indeterminate for UIP and acute exacerbation. (**B.1**) HRCT image: reticular abnormalities with traction bronchiectasis and non-basal predominance (circle). (**B.2**) HRCT image in left decubitus lateral six months after the previous examination: multifocal areas of ground glass opacities, overlapped with the previous pattern of pulmonary fibrosis. (**C**) Probable UIP CT pattern. (**C.1**–**3**) HRCT images: reticular pattern with traction bronchiectasis (arrows in **C.2** and **C.3**) and a peripheral subpleural and basal predominance; peripheral reticulation is non-segmental (crossing fissures), heterogeneous, and present in upper lobes. Note that there is no honeycombing, and the UIP histological pattern was proven with pulmonary biopsy. (**D**) IPF and lung adenocarcinoma. (**A**,**D.1**) HRCT images: typical UIP CT asymmetrical pattern and lung mass in the apical segment of the left lower lobe (star in **D.2**).

1. According to the latest official evidence-based guideline for the diagnosis of IPF [4], this disease is associated with a histopathological and/or radiological pattern of UIP. The radiological UIP pattern was defined by the presence of a reticular pattern and honeycombing, with or without visible traction bronchiectasis, and with a peripheral subpleural and predominantly basal distribution, in the absence of findings inconsistent with UIP (extensive ground glass opacities, micronodules, air trapping, non-honeycomb cysts, consolidation, or a non-basal or non-peripheral predominant distribution). Consensus has established three radiological patterns that IPF can present in HRCT: UIP, possible UIP (UIP-like pattern, but without honeycombing), and inconsistent with UIP (with non-peripheral or non-basal subpleural distribution, or with any finding inconsistent with UIP). Furthermore, it has been established that, in a patient with the appropriate clinical context, the presence of a UIP pattern in HRCT in the absence of a known cause for lung fibrosis (e.g., occupational and environmental exposures, systemic autoimmune disease, or drug toxicity) leads to an IPF diagnosis without the need to perform a lung biopsy, as the probability for such a pattern corresponding to a histopathological UIP pattern is 90–100% [16] (positive predictive value 97.3%, 95% confidence interval 92.3–99.4, in a subsequent study by Raghu et al. 2014 [17]). One of the key findings of this pattern is the honeycombing, which is an indicator of end-stage pulmonary fibrosis. The radiological UIP CT pattern allows, therefore, an IPF diagnosis in the advanced stages of the disease and is observed in less than 50% of patients. When the two other patterns appear in HRCT (possible UIP and inconsistent with UIP), according to the guidelines, it is necessary to do a pulmonary biopsy, and the final diagnosis will depend upon the combination of the different radio-pathological findings.
2. Recently, in 2018, the Fleischner Society published the latest IPF diagnostic criteria based on bibliographic review and experts’ opinions [5]. Four types of patterns can be observed as follows:
  - **Typical UIP CT pattern**: Reticular opacities and honeycombing, with peripheral traction bronchiectasis and subpleural and basal-predominant distribution. Furthermore, there must be no other findings suggesting an alternative diagnosis (see below, Figure 2A). The fibrosis distribution can be asymmetric. This corresponds to the 2011 UIP pattern guidelines. To make an IPF diagnosis, a lung biopsy is not required in the correct clinical context and in the absence of a known cause of pulmonary fibrosis.
  - **Probable UIP CT pattern**: Replaces the possible UIP pattern in the 2011 guidelines. It includes the same findings as the UIP pattern, although without honeycombing. A lung biopsy is not required in the correct clinical context and in the absence of a known cause of pulmonary fibrosis.
  - **CT pattern indeterminate for UIP**: Appears when the fibrosis presents a variable or diffuse distribution or when there are inconspicuous findings suggesting a non-UIP pattern (Figure 2B). A lung biopsy is required to diagnose IPF in these patients.
  - **CT features most consistent with a non-IPF diagnosis**: Appears when the pulmonary fibrosis is predominant in the upper or middle areas, it is peribronchovascular, it respects the subpleural area, or in any of the following features: predominant consolidation, extensive ground glass opacity without acute exacerbation, extensive mosaic pattern with air trapping on expiration, and nodules or cysts other than in a honeycomb formation. For example, fibrosis with peribronchovascular distribution predominantly in the upper areas, ground glass opacity, and air trapping in non-fibrotic areas, all suggest fibrotic hypersensitivity pneumonitis.

Other findings visible in HRCT are micro-calcifications overlapped with the fibrosis areas—more frequent in the UIP pattern—and mild mediastinal lymphadenopathies.

#### 1.2.2. Difficulties Interpreting the High-Resolution Computed Tomography

The 2011 guidelines gave great diagnostic relevance to the HRCT pattern and, especially, to the presence of honeycombing as the finding that could be used to identify the group of patients who, under specific clinic and radiologic characteristics, could be diagnosed with IPF without the need to undergo a surgical lung biopsy. However, the detection of honeycombing and its differentiation from other types of features is not always easy.

According to the Fleischner Society [18], honeycombing in HRCT consists of the presence of clustered cystic airspaces with well-defined walls and similar diameters between 3 and 10 mm (although possibly as large as 2.5 cm), which are usually subpleurally located. The guidelines do not specify if the honeycombing distribution can be in one or multiple layers, although some authors consider that a multi-layered distribution increases the diagnostic safety. The guidelines also do not indicate the typical thickness of the cyst wall in HRCT, although the histologic definition indeed specifies that they are cystic airspaces with fibrous, thick walls.

In HRCT, the honeycomb cysts walls are well defined, are shared, and can have variable thicknesses: thicker than the emphysema and bullas and thinner in larger cysts [19,20].

Honeycombing visualization is one of the major challenges in the characterization of pulmonary fibrosis, hence the agreement between multiple observers can be low even among expert thoracic radiologists. In a paper by Walsh et al., the agreement between multiple observers to detect the UIP CT pattern following the 2011 ATS/ERS/JRS/ALAT guidelines was only moderate [21]. In another study by Watadani et al. [22], there was no consensus in the identification of honeycombing in 29% of the cases, with the main causes of confusion being traction bronchiectasis and subpleural localized emphysema.

The distinction between honeycombing and traction bronchiectasis can be almost impossible in sequential HRCT studies with 1 mm axial slices every 10 mm. In these studies, rounded air images with thick walls corresponding to bronchiectasis and bronchiolectasis can be indistinguishable from honeycombing when seen tangentially, especially when they are grouped and have subpleural location. The distinction can also be difficult with volumetric HRCT if only axial images are considered, although the visualization of the continuity of air images helps to identify bronchiectasis. Useful reconstructions to differentiate honeycombing from traction bronchiectasis are thin-section multiplanar reformations and minimum intensity projection (minIP) reformations, as they allow the observation of the dilated and beaded tubular morphology, which is typical of the traction bronchiectasis [15]. Nevertheless, in many cases, it is impossible to differentiate between both structures, and what is interpreted as honeycombing in HRCT is a mixture of honeycombing, traction bronchiectasis, and bronchiolectasis, as has been proven in studies of radiologic–pathologic correlation [23].

From a histology point of view, microscopic honeycombing corresponds to a dilation of terminal airways (alveolar ducts and lumen) for collapsing of multiple fibrotic alveoli; these enlarged airspaces are surrounded by fibrosis, lined by bronchiolar or hyperplastic epithelium, and typically contain mucus. Their small size is below the HRCT resolution limit, so they are not visualized as cysts but as a reticular pattern. The end-stage of this process—when the cysts increase their sizes—radiologically corresponds to honeycombing that initially appears in the subpleural areas of the lower lobes.

The second cause for honeycombing confusion is emphysema. According to the Fleischner Society [18], paraseptal emphysema is characterized by subpleural and peribronchovascular areas of low attenuation separated by intact interlobular septa, sometimes associated with bullae. Bullae are airspaces that in HRCT appear as rounded areas with low attenuation, 1 cm or more in diameter, and a thin wall. Both paraseptal emphysema and subpleural bullae can be confused with honeycombing. Findings that help their differentiation are multi-layered disposition and a thicker wall of honeycombing, with the assessment being more difficult when there is a single layer of cysts. Furthermore, honeycombing is associated with traction bronchiectasis, which cannot be found in areas with emphysema or bullae. Multiplanar minIP reconstructions are very useful to evaluate the low-attenuating areas, the thickness of their wall, and the presence or absence of associated traction bronchiectasis. The differentiation between honeycombing and emphysema can be especially difficult or even impossible in smokers and former smokers with combined pulmonary fibrosis and emphysema in the areas of the lung where both processes coexist.

Another source of error in the detection of honeycombing is the overlapping of consolidation or ground glass attenuation areas—due to infectious or inflammatory processes—over lung emphysema, generating the false appearance of honeycombing.

Finally, honeycombing must be differentiated from other types of cysts, such as the airspace enlargement with fibrosis that are part of the smoking-related interstitial fibrosis spectrum, pulmonary Langerhans cell histiocytosis, lymphangioleiomyomatosis, lymphoid interstitial pneumonia, and other cystic diseases. The morphology and distribution of the features generated by these diseases help their differentiation.

#### 1.2.3. From Possible Usual Interstitial Pneumonia Pattern (ATS/ERS/JRS/ALAT 2011) to Probable Usual Interstitial Pneumonia Pattern (Fleischner 2018) in High-Resolution Computed Tomography

Studies subsequent to the publication of the 2011 guidelines have shown that the possibility of a patient with a possible UIP CT pattern—without or with little evidence of honeycombing—having a histopathological UIP pattern is very high. A paper containing results from the ARTEMIS-IPF study (made with patients having a UIP CT pattern with 5% or less honeycombing) showed that 79 out of 84 patients with a possible UIP CT pattern had a histopathological UIP pattern in pulmonary biopsy (positive predictive value 94%, confidence interval 95%: 86.7–98%) [17].

The pre-test probability of a histopathological UIP pattern in patients with possible UIP CT pattern increases with age (≥60-year-old), male sex, and presence of traction bronchiectasis, and it is independent of a honeycombing presence [24].

In patients with a possible UIP CT pattern, it has been observed that the disease progresses the same way as in patients with a UIP CT pattern treated with the same antifibrotic [25,26].

In 2013, Gruden et al. [27] identified the HRCT findings—other than honeycombing—that appeared in patients with a biopsy-proved UIP. Subsequently [28], these findings were validated in a prospective cohort of 38 patients, with long-term clinical tracking. According to these authors, UIP diagnostic criteria in HRCT—in the absence of honeycombing—are as follows: (i) peripheral reticulation with lobular distortion, (ii) traction bronchiectasis and bronchiolectasis with or without honeycombing, (iii) basal predominance even with some affectation of the upper lobes, (iv) non-segmentary (i.e., without respecting the fissures) and heterogeneous (i.e., patched, alternating non-affected areas and correlated to the temporal and spatial heterogeneity of the histopathological UIP pattern), and (v) the absence of inconsistent findings, asymmetrical but non-unilateral (Figure 2C). The distinction between honeycombing and traction bronchiectasis does not affect the diagnostic accuracy of the CT if the criteria above are strictly verified. Therefore, the authors proposed that the IPF consensus guidelines should change the “honeycombing with or without traction bronchiectasis” criterion into “traction bronchiectasis with or without honeycombing”. This should allow more agreement across multiple observers, an earlier diagnosis of the disease—instead of the honeycombing in the latest stage as is the case when there is a UIP pattern—its applicability to patients whose comorbidities sometimes make surgical lung biopsy unavailable, and the possibility of benefiting from new antifibrotic drugs potentially effective in slowing fibrosis progression.

The increasing importance of visible traction bronchiectasis in HRCT for the diagnosis of IPF is noticeable. Honeycombing and traction bronchiectasis/bronchiolectasis are considered part of the same process, unique and continuous, of bronchiolar dysplastic proliferation and aberrant lung remodeling, so-called bronchiolization of alveolar spaces, for which differentiation is currently somehow arbitrary. Some studies have shown that honeycombing diminishes in expiration, proving its relationship with the airway [29]. Micro-CT studies in explanted lungs [30] have proven that, in honeycombing, cysts sometimes connect among them and are in continuity with the bronchial tree.

In the initial phase of histological micro-honeycombing, with microcystic airspaces lined by bronchiolar epithelium, bronchiolization in HRCT can be seen in a reticular pattern and traction bronchiectasis, without radiological honeycombing. In the advanced phase of histological honeycombing, there is honeycombing in HRCT corresponding to cystic airspaces lined by bronchiolar epithelium, traction bronchiectasis, and bronchiolectasis.

Therefore, a new guideline is necessary based on evidence with accurate criteria for noninvasive diagnosis of patients with suspected IPF in the early stages of the disease and where it may not be possible to do a pulmonary biopsy.

#### 1.2.4. Identification of Comorbidities and Complications of Idiopathic Pulmonary Fibrosis in High-Resolution Computed Tomography

Other chronic fibrosing lung diseases can also appear and include combined pulmonary fibrosis and emphysema in smokers and former smokers, such as pulmonary hypertension, spontaneous pneumothorax, lung cancer, and acute exacerbation of IPF.

Combined pulmonary fibrosis and emphysema in smokers and former smokers consists in HRCT of the presence of paraseptal or centrilobular emphysema, predominantly in the upper lobes, and pulmonary fibrosis, predominantly in the lower lobes, although there is not yet a consensus definition of this clinical entity [31]. It is associated with pulmonary hypertension more frequently than IPF without emphysema.

Spontaneous pneumothorax is due to the rupture of a subpleural honeycomb cyst into the pleural space.

Presence of lung cancer can be suspected when a nodule or mass is observed in patients with pulmonary fibrosis, usually by the thickness of the fibrosis. The most frequent type is the adenocarcinoma, and the most frequent location is the lower lobes (Figure 2D) [32].

Acute exacerbation of IPF is an acute episode of clinical and functional respiratory worsening, with less than one month of evolution, with the presence in HRCT of bilateral ground glass opacities and/or consolidations over the underlying fibrosis pattern [33], in the absence of cardiac failure or fluid overload. It can appear in patients with known IPF or without a previous IPF diagnosis. It is due to the apparition of a histologic pattern of diffuse alveolar damage or organizing pneumonia over its histopathological UIP pattern. The cause can be unknown or there can be triggering factors, such as infection, aspiration, drugs, massive transfusion, and thoracic surgery, including the realization of a pulmonary biopsy or bronchoscopy. Although chest X-rays can show bilateral consolidations, patient evaluation must be made using HRCT whenever possible. In HRCT, areas of ground glass opacities, with or without consolidations, peripherical, multifocal, or diffuse, overlapped with the previous patterns of pulmonary fibrosis can be observed [34]. Radiologically, pulmonary causes of acute exacerbation, such as infection, extra-pulmonary and pulmonary embolism (which is the reason that HRCT must be done with endovenous contrast and angiographic techniques), pneumothorax, and pleural effusion, should be excluded. The prognostic is poor, with 50% patient mortality [35].

### 1.3. Histhological Diagnosis of Idiopathic Pulmonary Fibrosis

According to the Fleischner Society [5], surgical lung biopsies should be considered only in patients with indeterminate or inconsistent with UIP changes in HRCT or those with a clinical suspicion of an alternative diagnosis. In those with probable UIP, radiological patterns with high clinical suspicions of IPF lung biopsy could be avoided, because the predictive positive value of a histological specimen to diagnose a UIP histological pattern is over 94%.

Surgical lung biopsies (SLB) are considered the gold standard procedure to assess lung tissue specimens. They have shown a good diagnostic yield, reaching figures between 77% and 95% in most publications. Practical issues concerning SLB are that it should be taken from multiple lobes, avoiding the most severely affected ones; the lung tissue specimen should be 2 × 3 cm along the pleural margin and 1–2 cm deep.

Hutchinson et al. [36] noticed that less than 5% of patients with IPF underwent a SLB in in actual clinical practice in the UK. In high-risk patients for whom SLB is unavailable or in those who refuse the procedure, BAL or transbronchial biopsy (TBB) should be performed to rule out alternative disorders, according to the advice of the Fleischner Society [5]. The histological findings of BAL in IPF patients are nonspecific. There is an increased level of leucocytes or eosinophils count. BAL potential diagnostic utility lies in the detection of lymphocytosis. Particularly, levels above 30% in non-smokers or more than 20% in smokers [10,11], may suggest other IPF-mimicking fibrotic diseases, such as HP. The diagnostic yield of TBB in ILD is less than 35%. Transbronchial cryobiopsy (TBCB) is an emerging procedure with better diagnostic yield than conventional TBB (74% vs. 34%) but has an increased number of hemorrhages (56% vs. 34.2%) and pneumothorax (2–33%) [37]. Tomasetti et al. [38] reported that information obtained for SLB and TBCB had the same impact in the MDT, with a global diagnostic agreement of 0.73 with the TBCB and 0.86 with the SLB. The main difference was that the pathologist kappa index of cryobiopsy was lower than that of surgical biopsy (0.59 vs. 0.86). The main problem with TBCB is that the procedure lacks standardization and is only available in a few hospitals.

Usual interstitial pneumonia (UIP) is the characteristic pathological abnormality in an IPF [39] patient, but it is not pathognomonic and could be found in other ILDs described later [40,41,42,43]. The histological hallmark includes a heterogeneous appearance with alternating areas of normal lung, fibrosis, and honeycomb change. The fibrotic zones are composed mainly of dense collagen, with characteristic foci of proliferating fibroblasts. If any of these four criteria are missing, the accuracy of the IPF diagnosis is lower, particularly if they are combined with a non-UIP pattern in HRCT. IPF may only be diagnosed if a histological UIP pattern/probable UIP is found in the absence of a known cause or other histological features, suggesting an alternative diagnosis (Table 1). The Fleischner Society has proposed some changes to the traditional histological UIP patterns that are detailed in Table 1.

## 2. Differential Diagnosis

Interstitial lung diseases are a heterogeneous group of disorders that share similar clinical, radiologic, and pathologic characteristics; therefore, the diagnosis of IPF is often challenging. The prognosis and therapeutic options vary among the different causes and types of ILD, so ascertaining the correct diagnosis is crucial.

The MDT plays a central role in addressing a profound discussion of complex data, because so-called typical patterns are uncommon. Before considering IPF, several entities that can manifest a UIP pattern should be ruled out. This is also a laborious matter, because some diseases are not easily excluded, and some known causes of ILD are often unclear.

ILDs are divided into idiopathic interstitial pneumonia and those entities that are associated with known causes.

### 2.1. Differential Diagnosis with Other Idiopathic Interstitial Pneumonias

Classification of idiopathic interstitial pneumonias (IIP) has been a controversial topic. Several schemes have been published throughout the last years, and some entities have been included or eliminated. In 2013, an ATS and ERS classification consensus was published. ILDs were divided into major IIP, rare IIP, and unclassifiable IIP [44] (Table 2).

#### 2.1.1. Idiopathic Nonspecific Interstitial Pneumonia

This pattern can be found not only as an idiopathic condition but also secondarily as other disorders, such as SAD or toxicities. It was described in 1998 by Katzenstein et al. as a pattern that could not be classified as IPF, desquamative interstitial pneumonia (DIP), or Acute interstitial pneumonia (AIP) [39]. Finally, it was accepted as a distinct entity among the IIPs. Some important clinical characteristics that distinguish idiopathic nonspecific interstitial pneumonia (NSIP) from UIP are a subacute, rather than insidious onset of symptoms, and a lack of a strong male predominance. The most common radiological abnormalities in NSIP are bilateral ground glass opacities, traction bronchiectasis, and bronchiolectasis, which are found in approximately 75% of cases [45]. Honeycombing is usually absent at presentation but may increase in prevalence and extent during follow-up [46].

Histological findings are variable. They include varying amounts of interstitial inflammation and fibrosis with a uniform appearance, in contrast with the temporal heterogeneity found in UIP. The main change is an interstitial pneumonia characterized by expansion of alveolar septa by a variably dense infiltrate of predominantly mononuclear inflammatory cells with or without associated fibrosis [40,47]. The main features that are typical in UIP and should not be found in NSIP are patchy involvement and fibrosis, characterized by abrupt transitions, resulting in a patchwork pattern, fibroblast foci, and architectural distortion that include honeycomb change and/or interstitial scarring [41].

The overall prognosis is variable but is usually better than that of UIP. Some patients may respond to medical therapy, but a 15–26% mortality rate at five years is described [48].

#### 2.1.2. Smoking-Related Idiopathic Interstitial Pneumonia’s

Tobacco use is involved in the pathologic process of respiratory bronchiolitis–interstitial lung disease (RBILD) and DIP. The main feature is pigmented macrophage accumulation, with the distinction dependent on the extent and distribution of this process. They share several overlapping clinicopathological features, and both have a better prognosis than that of UIP.

#### 2.1.3. Respiratory Bronchiolitis–Interstitial Lung Disease

Histologic RBILD is often present in current smokers [45] and can be viewed as a physiological response to smoking, which, in a few individuals, becomes extensive enough to result in an interstitial lung disease. The clinical features of RBILD are nonspecific. It typically occurs during the fourth or fifth decades of life and almost always affects current or former heavy smokers [49].

The characteristic radiologic features are ground glass opacity and centrilobular nodules.

The main pathologic finding is the presence of pigmented macrophages within the lumen of respiratory bronchioles, accompanied by a patchy submucosal and peribronchiolar infiltrate of lymphocytes and histiocytes. These smokers´ macrophages can be also found in bronchoalveolar lavage in the absence of lymphocytosis [50].

The disease course is heterogeneous. Survival is common, but symptomatic and physiologic improvement occurs in only a minority of patients, and neither smoking cessation nor immunosuppressive therapy is regularly associated with clinically significant benefit [51].

#### 2.1.4. Desquamative Interstitial Pneumonia

DIP is an uncommon cause of IIP. It usually affects cigarette smokers in their fourth or fifth decades of life; however, a small percentage of cases can be seen in non-smokers, usually related to connective tissue disorders, drug toxicities, or viral infections.

HRCT shows ground glass opacities, usually symmetrical, without the peripheral reticular and reticulonodular opacities characteristic of UIP [50,52].

Histologically, the presence of numerous mononuclear cells within most of the distal airspaces is characteristic. Alveolar septa are thickened by a sparse inflammatory infiltrate that often includes plasma cells and occasional eosinophils [50].

Smoking cessation is a key component of the management of DIP, although the exact impact of smoking cessation is unclear. Glucocorticoid therapy is sometimes used with variable outcomes [50,52].

#### 2.1.5. Acute or Subacute Idiopathic Interstitial Pneumonia’s

##### Cryptogenic Organizing Pneumonia

Cryptogenic organizing pneumonia (COP) usually affects patients in the fifth or sixth decade, predominantly in former smokers or non-smokers. Clinically, it presents as a subacute nonspecific illness, with cough, weight loss, fever, and dyspnea.

HRCT characteristically demonstrates ground glass opacity associated with patchy and often migratory consolidation in a subpleural, peribronchial, or bandlike pattern. Consolidation may be associated with partial or complete resolution, whereas reticular opacity is associated with persistent or progressive disease [44].

Typical histologic findings are excessive proliferation of granulation tissue, which consists of loose collagen-embedded fibroblasts and myofibroblasts, involving alveolar ducts and alveoli, with or without bronchiolar intraluminal polyps.

Oral corticosteroid therapy is often successful, but relapse is common.

##### Acute Interstitial Pneumonia

AIP is a severe, rapidly progressive form of IIP. The short-term mortality rate is 50% or more, and there is no proven treatment. It usually affects previously healthy individuals who develop rapidly progressive respiratory failure.

In the early stage, HRCT shows bilateral patchy ground glass opacities, often with consolidation of the dependent lung. Later, distortion of bronchovascular bundles and traction bronchiectasis can be found [53].

Histologically, the main finding is an organizing or proliferative stage of diffuse alveolar damage (DAD). The key features include diffuse distribution, loose organizing connective tissue causing alveolar wall thickening, and prominent pneumocyte hyperplasia [44].

#### 2.1.6. Rare Idiopathic Interstitial Pneumonia’s

##### Idiopathic Lymphoid Interstitial Pneumonia

Idiopathic lymphoid interstitial Pneumonia (ILIP) is one entity within a spectrum of lymphoproliferative disorders that can involve the lung. Most cases are associated with other diseases, but primary cases still occur [54].

The radiologic findings are similar to those seen in NSIP, such as ground glass attenuation, centrilobular nodules, and interstitial thickening, with a lower lobe predominance. In contrast to NSIP, lung cysts are frequent [44,54].

The most common pathologic findings are extensive alveolar septal infiltration of lymphocytes, plasma cells, and histiocytes [54].

##### Idiopathic Pleuroparenchymal Fibroelastosis

Idiopathic pleuroparenchymal fibroelastosis (IPPFE) consists of fibrosis involving the pleura and subpleural lung parenchyma, predominantly in the upper lobes. Clinically, shortness of breath, recurrent respiratory infections, and pneumothorax are common. Nonspecific auto-antibodies and coexistence with other ILDs may be present.

HRCT shows upper pleural thickening and subpleural consolidation with traction bronchiectasis and loss of volume. Subpleural cysts are sometimes seen. 

Histologic findings are intra-alveolar fibrosis with septal elastosis, pleural fibrosis, and nonspecific chronic inflammation, focally with lymphoid follicle formation [44].

The prognosis is poor, with no proven treatment.

### 2.2. Differential Diagnosis with Known Causes of Usual Interstitial Pneumonia Pattern

The exclusion of other known conditions with a UIP pattern (Table 3) has serious therapeutic and prognostic implications.

#### 2.2.1. Chronic Hypersensitivity Pneumonitis

IPF and chronic hypersensitivity pneumonitis (CHP) can be indistinguishable, and the latter can be misdiagnosed. Patients usually forget or even have no idea of having been exposed to antigens (especially when it occurs in an indirect way), making the causative agent unidentifiable [10,11]. A thorough clinical history is paramount. Dedicated questionnaires have been proposed but have not been validated.

Laboratory tests include the detection precipitins or specific IgG but may be confusing, as positivity indicates exposure but not necessarily disease. Negativity is not discriminatory, as they vanish with time. Acropachies that are frequently in IPF are seen in up to 50% of CHP patients [1,3].

Hypersensitivity pneumonitis was classically classified into acute, subacute, and chronic forms, with the latter being the most challenging. Recently, Varacova and Salisbury [10,11] have proposed a new classification more in line with the prognosis of the disease: acute or inflammatory HP and chronic fibrotic HP, avoiding the term “subacute.” Honeycombing is as frequent in CHP as in IPF, although the former has a patchy mid-upper zone predominance [10,11]. Ground glass opacities and mosaic attenuation indicating air trapping are clues to differentiate CHP from IPF. Anyway, radiological features can be indistinguishable in some patients [11].

The pathological pattern can be very similar in both conditions, but fibroblastic foci are less predominant in CHP and are usually found surrounding respiratory bronchioles [51]. Some features, such as the presence of bridging fibrosis (that can also not be seen in asbestosis), intraluminal fibrosis, isolated giant cells or granulomas, lymphoid aggregates, and centrilobular fibrosis are red flags for CHP diagnosis [55].

#### 2.2.2. Connective Tissue Disease in Interstitial Lung Diseases

Connective tissue diseasae (CTD) patients are at risk of ILDs. The most common are rheumatoid arthritis (RA), systemic sclerosis (SSc), and antisynthetase syndrome. Idiopathic interstitial pneumonias develop a CTD in 15% of cases [56]. CTD-ILD has a better prognosis and a different management (immunosuppressant therapy) than IPF.

Three possible scenarios can make diagnosis challenging: (1) the combination of both diseases; (2) ILD preceding CTD by many years (e.g., inflammatory myopathy, RA, and SSc although in the latter is rare) [13]; and (3) ILD having CTD features without diagnostic criteria.

CTD and ILD combination has some aspects that must be kept in mind when assessing CTD as follows:

- Epidemiology: CTDs have are more common in non-smokers and young women with a NSIP pattern [42]. RA is an exception, affecting mostly elderly, smokers, and men. A NIU pattern in a young (<50 year-old) woman should prompt the suspicion of an occult CTD.
- Radiology: NSIP is the most frequent pattern [56]. However, a UIP pattern can be found, especially in an established CTD, such as RA and SSc. Rheumatoid arthritis, remarkably, lacks honeycombing [57]. The HRCT RA-UIP pattern has a worse survival than RA-NSIP. The HRCT RA-UIP pattern has a worse survival than RA-NSIP.
- Histology: RA-UIP has less fibroblast foci, smaller honeycombing cysts, and more pronounced lymphoid hyperplasia than IPF-UIP [57]. Lymphoid aggregates with germinal centers have statistical significance. SSc-related fibrosis has temporal and spatial homogeneity, possibly being pleural fibrosis as well. Lymphocytic infiltrates without fibrosis appear in less than 10% of SSc patients. Lymphocytic interstitial infiltrate is more prominent in Sjögren syndrome.

A most challenging scenario is to distinguish IPF from changes secondary to CTD when the rheumatology disease does not fulfill diagnostic criteria or is occult.

Autoantibodies, such as ANA and RF are frequently positive low titers but lack clinical significance. ATS/ERS/JRS have recommended testing for autoantibodies in IPF assessment [3]. It has been stated that IPF with positive antibodies made its histology more similar to that of CTD with the presence of more germinal centers and plasmatic cells [57]. Their prognostic value is controversial [58]. In contrast, high titers of ANA (1:320) or the positivity of more specific autoantibodies, such as anti-SS-A, anti-CCP [9], and ANA with nucleolar or centromere patterns [13] are predictors of future CTD development.

Patients having a ‘flavor’ of CTD but not conforming to diagnosis criteria have been named in many different ways: undifferentiated CTD (UCTD), lung dominant CTD and autoimmune feature interstitial lung disease (AFI-ILD). Furthermore, diagnostic criteria are inconsistent between reports. UCTD is mainly present in NSIP as opposed to IPF patients. To further complicate things, UCTD diagnostic criteria differs from paper to paper, which makes incidence variable between studies [13,57]. The most common radiologic pattern associated with these conditions is the NSIP pattern, although UIP has been reported [13]. In 2015, a working group on behalf of ATS/ERS was created to adapt nomenclature and unify criteria [13]. Ultimately, this category was named interstitial pneumonia with autoimmune features (IPAF). Clinical, serological (with specific autoantibodies), and morphological domains (histology and radiology of CTD) were coined. One feature of at least two domains needs to be present for diagnosis. The UIP pattern is not included in either radiological nor histological domains. As the authors explain, the UIP pattern by itself does not increase the possibility of CTD development in the future. Nevertheless, overlap between UIP and NSIP or COP patterns have been described. Nowadays, IPAF is not a validated term.

#### 2.2.3. Exposure-Related Lung Disease: Asbestosis

Asbestos fiber inhalation, whether occupational or domestic, may lead to asbestosis and other lung diseases. Diagnosis has important legal and economic implications. There is a dose-response relationship. Asbestosis develops after a significant exposure of at least several months. Minimal doses may produce radiological changes, such as pleural plaques [59], otherwise very uncommon in IPF.

Asbestosis has a slow progression and tends to stabilize, although accelerated forms similar to IPF have been described. Diagnosis is usually made by exposure history and radiology [43].

HRCT features in advanced stages overlap those of IPF. Periphery and lower lobe preference are similar in both entities, but fibrosis is coarser in asbestosis. Subpleural dots that may coalesce to form subpleural lines are more frequent in early stages. The former may be found in less fibrotic areas in advanced disease [59]. Pleural thickening, visceral and parietal, is a good clue to differentiate asbestosis from IPF. While parenchymal bands are related to visceral pleural affectation, pleural plaques are associated with parietal pleura [43].

Histologic diagnosis has two well-stablished criteria: diffuse interstitial fibrosis and asbestos bodies. The latter indicates severity and exposure when isolated [43]. Fibrotic UIP or NSIP patterns can be found. The former has a basal predominance with more distance from pleura and shows temporal and spatial homogeneity [59]. In early stages, fibrosis is located in the walls surrounding the bronchioles. Fibroblastic foci are very rare, and when numerous, other conditions have to be suspected. Whether a UIP pattern found in biopsy is an atypical form of asbestosis or IPF is often a topic of discussion. The fact that patients with asbestos exposure and UIP pattern did not have a dose-response disease was demonstrated by Atanoos et al. [60].

#### 2.2.4. Drug-Related and Radiation-Related Lung Disease

In these conditions, clinical and radiologic manifestations usually reflect the underlying histopathologic mechanism: cytotoxic (as bleomycin) and noncytotoxic (as amiodarone). Differential diagnosis is challenging, and they are frequently misdiagnosed because of the lack of relationship between exposure and the onset of disease. Some of them can occur years after discontinuation. Furthermore, many drugs can cause acute, subacute, or chronic pulmonary toxicity. The most common radiological patterns are NSIP, organizing pneumonia, and DAD, although pulmonary fibrosis can be seen as well Drug discontinuation and, in some cases, the aid of oral corticosteroids are necessary [61]. Pneumotox.com website may be useful tool to look for a potential harmful drug.

Radiotherapy can cause pulmonary fibrosis as well, and it is an inevitable effect [62]. The risk factors are age and previous chemotherapy and radiotherapy techniques. Lung cancer patients are more affected than breast cancer patients. Toxicity can be divided into acute (radiation pneumonitis) and late (pulmonary fibrosis), the latter occurring after six months of treatment discontinuation [58]. General supportive management, oxygen when needed, and, in some cases, oral corticosteroids are therapeutic options.

#### 2.2.5. Microaspiration-Related Lung Disease: Gastroesophageal Reflux

Aspiration of foreign material into airways leads to a broad spectrum of disease, such as chronic lipoid pneumonia, obliterative bronchiolitis, and diffuse aspiration bronchiolitis [63]. This syndrome depends on several factors, including the nature, quantity, and frequency of the aspirated material and the host’s response to injury. Non-erosive gastroesophageal reflux (NERD) and gastroesophageal reflux disease (GERD) are the most relevant in the differential diagnosis of IPF, as gastroesophageal reflux (GER) is present in up to 90% of patients with IPF. It is not always mean microaspiration. Silent aspiration (asymptomatic GER) supposes a challenge for clinicians as occult aspiration has been suggested as a trigger to acute IPF exacerbation. Several experimental studies suggest microaspiration as a potential cause of pulmonary fibrosis, especially in animal models [63]. The risk factors include depressed consciousness, esophageal dysfunction, dysphagia due to neurologic disorders, compromised airways defenses, reflux, obesity, and obstructive sleep apnea [63]. Diagnosis requires 24-h pH monitoring and pH-impedance testing.

High-Resolution Computed Tomography shows reticulation with ground glass opacities and centrilobular nodules [63]. In more severe cases, pulmonary fibrosis is found.

Some pathologic criteria, such as exogenous lipoid interstitial pneumonia, poorly formed granulomas, and body-type multinucleated giant cells need to be present. BAL shows high levels of pepsin and bile acid [63].

Whether microaspiration is a single entity or only a risk factor for pulmonary fibrosis needs to be elucidated.

#### 2.2.6. Hermansky–Pudlak Syndrome

Hermansky–Pudlak syndrome (HPS) is a rare autosomal recessive disease. The estimated prevalence is 1:500,000–10,000,000 worldwide (1:1800 in Puerto Rico). Different mutations on the large arm of chromosome 10 leads to liposomal accumulation of ceroid–lipofuscin [64]. Oculocutaneous hypopigmentation (albinism), platelet dysfunction, granulomatous colitis, and, in some subtypes, pulmonary fibrosis (PF) are all characteristics of the disease. Nine types have been described with HPS1 being the most severe and most commonly associated with PF, with this one being the leading cause of mortality. The HPS4 subtype can lead to PF as well [64]. Women and Puerto Ricans in their third–fourth decades are more frequently affected. PF and IPF of HPS are very similar, although some differences exist.

Tomography shows subtle reticulation, interlobular septal and pleural thickening, ground glass opacities, and peripheral honeycombing in early stages. More advanced cases are associated with a coarser reticulation, peribronchovascular thickening, subpleural cysts, and central airways bronchiectasis. Mid-lower lobe predisposition and upper lobe honeycombing confirm HPS diagnosis. Pirfenidone might slow progression in severe lung pulmonary impairment. Lung transplant is ultimately required, although coagulation may be carefully assessed due to bleeding-related platelet dysfunction.

## 3. Conclusions

The diagnosis of IPF is a challenging, because there are no pathognomonic findings, and the main problem is to make a good differential diagnosis with other entities. The MDT, incorporating an expert pneumologist, radiologist, and pathologist, has become the new gold standard to diagnose and manage the disease. An MDT reduces diagnostic imprecision and increases the accuracy of the diagnosis by combining information from all three domains, even in less experienced centers.

## Figures and Tables

**Table 1:** Differences in Histologic criteria of UIP pattern between the American Thoracic Society (ATS), European Respiratory Society (ERS), Japanese Respiratory Society (JRS), and Latin American Thoracic Association (ALAT) guidelines 2011 [4].

ATS-ERS-JRS-ALAT Guideline 2011					
UIP Pattern (All Four Criteria)	Probable UIP	Possible UIP (All Three Criteria)		Not UIP Pattern (Any of Them)	
- Evidence of marked fibrosis/architectural distortion, honeycombing in a predominantly subpleural/paraseptal distribution - Presence of patchy involvement of lung parenchyma by fibrosis - Presence of FF - Absence of features against a diagnosis of UIP suggesting an alternate diagnosis.	- Evidence of marked fibrosis /architectural distortion, honeycombing - Absence of either patchy involvement or FF, but not both - Absence of features against a diagnosis of UIP suggesting an alternate diagnosis or Honeycomb changes only.	- Patchy or diffuse involvement of lung parenchyma by fibrosis, with or without interstitial inflammation - Absence of other criteria for UIP - Absence of features against a diagnosis of UIP suggesting an alternate diagnosis		- Hyaline membranes - Organizing pneumonia - Granulomas - Marked interstitial inflammatory cell infiltrate away from honeycombing - Predominant airway centered changes - Other features suggestive of an alternate diagnosis	
**Fleishner Society White Paper**					
**UIP-IPF Pattern (All Four Criteria)**	**Probable UIP-IPF (Not All Four Criteria)**			**Indeterminate for UIP-IPF**	**Features Most Consistent with an Alternative Diagnosis**
Patients show features with all four criteria, and do not show features that might suggest an alternative diagnosis (e.g., non-UIP)	Honeycomb fibrosis only or; dense fibrosis causing architecture remodeling with frequent honeycombing; patchy lung involvement by fibrosis; FF at the edge of dense scars may or may not be present		Possible-UIP disappears in Fleischner White paper	Less compelling histological changes than those classified by the final column (e.g., occasional foci of centrilobular injury or scarring, rare granulomas or giant cells, only a minor degree of lymphoid hyperplasia or diffuse inflammation, or diffuse homogenous fibrosis favouring fNSIP);	*Non-UIP pattern:* features of other fibrotic disorders—e.g., fHP, fNSIP, fOP, PPFE, pulmonary Langerhans cell histiocytosis, or smoking-related interstitial fibrosis; *UIP pattern with ancillary features strongly suggesting an alternative diagnosis:* e.g., prominent DAD or OP (consider acute exacerbation of UIP), granulomas, (consider HP, sarcoid, infection), marked interstitial inflammatory cell infiltrate away from areas of UIP (consider HP).

UIP: Usual Interstitial pneumonia histologic pattern; FF: fibroblast foci; fHP: hypersensitivity pneumonitis; fNSIP: fibrotic non-specific interstitial pneumonia; fOP: fibrosing organizing pneumonia; PPFE: pleuroparenchymal fibroelastosis; DAD: diffuse alveolar damage; OP: organizing Pneumonia; HP: hypersensitivity pneumonitis.

**Table 2:** Classification of idiopathic interstitial pneumonias. Modified from Travis et al. [44].

Unclassifiable Idiopathic Interstitial Pneumonias
Major Idiopathic Interstitial Pneumonias
Idiopathic pulmonary fibrosis (IPF)
Idiopathic nonspecific interstitial pneumonia (NSIP)
Respiratory bronchiolitis–interstitial lung disease (RBILD)
Desquamative interstitial pneumonia (DIP)
Acute interstitial pneumonia (AIP)
Cryptogenic organizing pneumonia (COP)
**Rare idiopathic interstitial pneumonias**
Idiopathic lymphoid interstitial pneumonia (ILIP)
Idiopathic pleuroparenchymal fibroelastosis (IPPFE)

**Table 3:** Known Causes with an UIP Pattern.

Chronic hypersensitivity pneumonitis (CHP)
Connective tissue disease (CTD)-related ILD
Exposure-related lung disease: Asbestosis
Drug-related and radiation-related lung disease
Microaspiration-related lung disease: gastroesophageal reflux (GER)
Hermansky–Pudlak syndrome
